# Antitumor Effects of Sesamin via the LincRNA-p21/STAT3 Axis in Human Bladder Cancer: Inhibition of Metastatic Progression and Enhanced Chemosensitivity

**DOI:** 10.7150/ijbs.103274

**Published:** 2025-03-31

**Authors:** Chao-Yen Ho, Thomas I-Sheng Hwang, Pei-Wen Peng, Te-Fu Tsai, Kuang-Yu Chou, Hung-En Chen, Peng-Hui Chang, Wei-Chien Huang, Chung-Hua Hsu, Tsai-Ju Chien, An-Chen Chang

**Affiliations:** 1 Institute of Traditional Medicine, School of Medicine, National Yang Ming Chiao Tung University, Taipei 112304, Taiwan, R.O.C.; 2 Division of Urology, Department of Surgery, Shin Kong Wu Ho-Su Memorial Hospital, Taipei 111045, Taiwan, R.O.C.; 3 Division of Urology, School of Medicine, Fu-Jen Catholic University, New Taipei 242062, Taiwan, R.O.C.; 4 Department of Urology, Taipei Medical University, Taipei 110301, Taiwan, R.O.C.; 5 School of Dental Technology, Taipei Medical University, Taipei 110301, Taiwan, R.O.C.; 6 Department of Urology, Wuri Lin Shin Hospital, Taichung 41454, Taiwan, R.O.C.; 7 Graduate Institute of Biomedical Sciences, China Medical University, Taichung 40402, Taiwan, R.O.C.; 8 Center for Molecular Medicine, China Medical University Hospital, Taichung 40447, Taiwan, R.O.C.; 9 Branch of Linsen, Chinese Medicine and Kunming, Taipei City Hospital, Taipei 11008, Taiwan, R.O.C.; 10 Division of Hemato-Oncology, Department of Internal Medicine, Branch of Zhongxing, Taipei City Hospital, Taipei 103212, Taiwan, R.O.C.; 11 Translational Medicine Center, Research Department, Shin Kong Wu Ho-Su Memorial Hospital, Taipei 111045, Taiwan, R.O.C.; 12 School of Oral Hygiene, College of Oral Medicine, Taipei Medical University, Taipei 110301, Taiwan, R.O.C.

**Keywords:** bladder cancer, sesamin, chemosensitivity, MMP2, LincRNA-p21

## Abstract

Bladder cancer (BC) ranks as the tenth most common malignancy worldwide, with high recurrence and progression rates despite current treatments. The matrix metalloproteinases (MMPs), particularly MMP2, play critical roles in tumor invasion and metastasis, contributing to poor prognosis. The p53-induced long noncoding RNA (lncRNA) lincRNA-p21, which acts as a tumor suppressor, has been implicated in various cancers, but its role in BC remains unclear. Sesamin, a bioactive lignan derived from sesame oil, has shown promise as a chemopreventive agent with multiple antitumor effects. In this study, sesamin was found to significantly inhibit cell viability *in vitro* and tumor formation *in vivo*. Additionally, sesamin inhibits MMP2 expression by downregulating the STAT3 signaling pathway, leading to reduced tumor cell migration, invasion, and anoikis resistance. LincRNA-p21 was identified as a crucial mediator in this process, helping sesamin reduce STAT3 activity. Co-administration of a PARP inhibitor with sesamin further enhanced the sensitivity of BC cells to conventional chemotherapeutic drugs (cisplatin, doxorubicin, epirubicin, mitomycin-c), suggesting its potential as an adjuvant therapy. These findings highlight the potential of sesamin as a therapeutic agent, both as a standalone treatment and in combination with conventional chemotherapy, to reduce tumor progression and chemotherapy-related toxicity in BC patients.

## Introduction

Bladder cancer (BC), which typically originates from the bladder urothelium, is the tenth most common malignancy globally, with approximately 82290 new cases and 16710 deaths reported in the United States in 2023 1. Around 75-85% of new BC cases are diagnosed as non-muscle-invasive bladder cancer (NMIBC). These cases are generally managed with transurethral resection of the tumor followed by postoperative intravesical therapy 2. Despite these treatments, NMIBC has a high postoperative recurrence rate of 78% and a progression rate of 45% 3. The lack of understanding of the molecular biological mechanisms underlying BC may lead to unsatisfactory therapeutic control. Identifying the driving factors and inhibitors is crucial for discovering new therapeutic targets.

Matrix metalloproteinases (MMPs), a family of zinc-containing endopeptidases, play a role in degrading the extracellular matrix (ECM). There are 26 known MMPs, which share many structural and functional similarities but differ in their substrate specificities 4. MMP2 and MMP9, belonging to the gelatinase group, contain fibronectin type II-like repeats within their catalytic domains and exhibit high affinity for gelatin and elastin 5. By breaking down the epithelial-mesenchymal transition (EMT) in the tumor microenvironment, MMP2 facilitates tumor cell invasion into adjacent tissues, promoting metastasis 6. Additionally, MMP2 releases various pro-angiogenic factors, which contribute to the blood supply needed for tumor growth. High expression of MMP-2 is generally indicative of a highly invasive tumor and is closely linked to metastasis and poor prognosis in breast cancer, gastric cancer, and BC as well.

Long non-coding RNAs (lncRNAs), which are transcripts longer than 200 nucleotides and belong to the ncRNA family, have demonstrated significant roles in the initiation and development of tumors 7. The elevated expression of lncRNA UCA1 has been positively associated with BC grade and is considered a novel diagnostic and prognostic marker 8, 9. Long intervening noncoding RNAs (lincRNAs), a subtype of lncRNAs, are defined as transcript units that discretely intervene between known protein-coding loci 9. A recent study has identified that the novel lincRNA-AATBC is significantly overexpressed in breast cancer tissues. The silencing of lincRNA-AATBC expression was found to induce apoptosis, primarily mediated through the activation of the JNK and NRF2 signaling pathways 10. LincRNA-p21, also known as TRP53COR1 (P53 Pathway Corepressor 1), has been associated with the progression of various cancers 11-15. However, its involvement in BC remains undetermined.

Sesamin, a lignan isolated from sesame oil, has demonstrated various bioactive effects, including immunomodulatory, anti-inflammatory, antitumor, and apoptotic properties 16, 17. As a potential chemopreventive agent, sesamin has been shown to inhibit proliferation in human hepatocellular carcinoma cell lines, regulate autophagy in colon cancer cells, and enhance apoptosis in human chronic myeloid leukemia cells 18. In this study, we investigated the anticancer properties of the health supplement sesamin: (1) Sesamin prevents the formation of bladder tumors; (2) Sesamin inhibits MMP2 expression by modulating the lincRNA-p21/STAT3 signaling pathway, thereby reducing key mechanisms of distant metastasis in bladder tumors, including migration, invasion, and anoikis resistance; (3) We also confirmed that sesamin can be used as an adjuvant therapy to enhance the sensitivity of conventional chemotherapeutic drugs, potentially allowing for reduced clinical dosages and minimizing chemotherapy side effects.

## Materials and Methods

### Cell culture

Human urinary tract epithelium cell line--SV-HUC-1 and human BC cell lines--T24, UMUC63, and 5637 were acquired from the Bioresource Collection and Research Center (BCRC, Hsinchu, Taiwan). Each of the following cell lines was cultured in its corresponding medium: SV-HUC-1 in F-12K (Gibco, Thermo Fisher Scientific, MA, USA); 5637 in Roswell Park Memorial Institute-1640 (RPMI-1640) (Gibco); T24 in McCoy's 5A (Gibco); and UMUC3 in Minimum Essential Medium (MEM) (Gibco). All media were supplemented with 10% fetal bovine serum (FBS), 2 mM GlutaMAX-1, 100 units/mL penicillin, and 100 μg/mL streptomycin, and maintained at 37°C in a humidified atmosphere with 5% CO_2_.

### Colony formation assay

BC cells were plated in six-well plates at a concentration of 3 × 10^5^ cells per well, where they were allowed to grow and form colonies, each consisting of at least 50 cells, over the course of a 7-day incubation period with sesamin and chemotherapeutic drug. The colonies were detected following the protocol outlined in our previous study 19.

### Wound healing assay

A wound healing assay was conducted using a three-well silicone insert (Ibidi GmbH, Gräfelfing, German) to create uniform scratches across the cell monolayer. BC cells were exposed to various concentrations of sesamin (0, 10, 30, and 50 µM) for 24 h. Post-treatment, non-adherent cells were gently washed away with PBS, and the remaining adherent cells were cultured in serum-free medium for an additional 24 h. Cell migration into the wound area was imaged using a Zeiss microscope. The gap width was measured at multiple defined points along the scratch using ImageJ software v1.44p (National Institutes of Health).

### Anoikis resistance assay

24-well plates were first coated with poly-HEMA (20 mg/mL in ethanol) to create a non-adhesive surface, and the plates were allowed to dry for 24 h at room temperature. UMUC3 cells were then seeded at a density of 3×10⁵ cells per well in MEM on these low-attachment plates to promote anoikis resistance. After 24 h of seeding, the cells were treated with sesamin at concentrations of 0, 10, 30, and 50 µM. At 2, 4, and 6 days post-treatment, the cell aggregates formed under non-adherent conditions were carefully collected. These aggregates were disaggregated into single-cell suspensions by gentle pipetting to ensure minimal cell damage. Cell viability was then assessed by staining the cells with a 0.4% trypan blue solution, and viable cells were counted using a hemocytometer.

### Transwell migration/invasion assay

To conduct cell migration and invasion assays, Transwell inserts (8 μm pore size; Costar, Corning, NY, USA) were employed in 24-well plates. For the invasion assay, the upper chamber was first coated with 30 μL of Corning Matrigel matrix (Corning, NY, USA) and allowed to set for 30 min. Subsequently, 1 × 10^4^ BC cells, suspended in 200 μL of serum-free medium, were introduced into the upper chamber, while the lower chamber was filled with 300 μL of medium containing 1% FBS. After a 24-h incubation period, the cells that had successfully migrated and invaded into the lower chamber were stained using 0.05% crystal violet, and the number of cells was then quantified.

### Western blot analysis

Total cell lysates were obtained using a radioimmunoprecipitation (RIPA) lysis buffer. Protein concentrations were quantified using the BCA Protein Assay Kit (Thermo Scientific, Waltham, MA, USA). Immobilon polyvinyl difluoride (PVDF) membranes were incubated with primary antibodies for 2 h, followed by incubation with horseradish peroxidase-conjugated anti-mouse or anti-rabbit secondary antibodies against N-cadherin (Abcam, CB, UK), MMP-2, MMP-9, MMP-11, VEGF-c, VEGF-a and GAPDH (GeneTex, CA, USA) for 1 h. Immunoreactive bands were visualized using the ImageQuantTM LAS 4000 biomolecular imager (GE Healthcare Life Sciences, Chicago, Illinois, USA).

### Immunofluorescence (IF) assay

BC cells were seeded onto a chamber slide (Sigma-Aldrich/Merck KGaA) and subjected to the sesamin treatments. The cells were then incubated for 1 h at room temperature with a primary antibody against STAT3, diluted 1:50 (Cell Signaling Technology, Danvers, MA, USA). After incubation, the cells were counterstained with 4′,6-diamidino-2-phenylindole (DAPI) for 5 minutes. The translocation of STAT3 from the cytoplasm to the nucleus in BC cells was subsequently visualized using a confocal microscope (Nikon, Tokyo, Japan).

### LincRNA-p21 Tet-On construction

The Tet-On inducible LincRNA-p21 expression construct was generously provided by Prof. Wei-Chien Huang from China Medical University 20. To create this construct, the full-length cDNA of LincRNA-p21 was amplified and cloned into the pAS4.1w.Ppuro-aOn vector (Addgene, Watertown, MA, USA), enabling inducible expression in the presence of tetracycline (10 μg/mL). The resulting pAS4.1w.Ppuro-aOn-LincRNA-p21 plasmid was then transfected into BC cells using the TransIT-LT1 transfection reagent (Mirus Bio, Madison, WI, USA), following the manufacturer's protocol. After 24 h, BC cells were treated with tetracycline (10 μg/mL) for an additional 24 h to induce LincRNA-p21 expression. Induction efficiency was confirmed by qRT-PCR.

### Human LncProfiler qPCR array

The Human LncRNA Profiler qPCR Array kit was purchased from System Biosciences (Palo Alto, CA, USA). To assess the expression of lncRNAs, BC cells (3 × 10^5^) were seeded onto a 6-well plate and treated with or without sesamin at a concentration of 50 µM for 24 h. A total of 90 lncRNAs were detected, following the manufacturer's protocol.

### Animal model

Six-week-old male BALB/c nude mice were acquired from BioLASCO Taiwan Co., Ltd. (Taipei, Taiwan) and maintained under specific pathogen-free conditions. All procedures of the animal study were approved by the Institutional Animal Care and Use Committee (Approval No. 112NSTCIACUC005) and performed according to the Guidelines of Animal Experimentation of Shin Kong Wu Ho-Su Memorial Hospital. Each mouse was subcutaneously inoculated with approximately 1×10^6^ UMUC3 cells. One week after inoculation, the mice were randomly divided into two groups: the control group, which received PBS injections, and the treatment group, which was administered sesamin at a dose of 10 mg/kg via intraperitoneal injection twice weekly for three weeks. Tumor growth was carefully monitored throughout the study by measuring both the volume and weight of the tumor tissues.

### Immunohistochemistry (IHC)

Paraffin-embedded tissues were collected from the animal model. Tumor tissues were then stained with anti-Ki-67 (Abcam, CB, UK), anti-MMP2 (GeneTex, CA, USA) or anti-PARP (Abcam, CB, UK) following the protocol outlined in our previous study 21.

### Database

The Cancer Genome Atlas Urothelial Bladder Carcinoma (TCGA-BLCA) dataset was used to analyze the expression of metastasis-related genes. Using the Gene Expression Omnibus (GEO) database (GDS1479 / 201069_at), we compared the expression levels of the indicated genes between superficial transitional cell carcinoma and muscle-invasive bladder cancer (MIBC) in patients with BC. The Human Protein Atlas was employed to examine MMP2 expression in human BC tissues.

### Statistical analysis

Each experiment was performed thrice in triplicate. Data are presented in terms of mean ± standard deviation (SD) values. Student's t test was used to compare mean values between two experimental groups, whereas one-way analysis of variance followed by Bonferroni post hoc test was used to compare mean values between >2 groups. Statistical significance was set at *P* < 0.05.

## Results

### Sesamin suppresses the survival of BC cells both in vitro and in vivo

**Figure 1:**
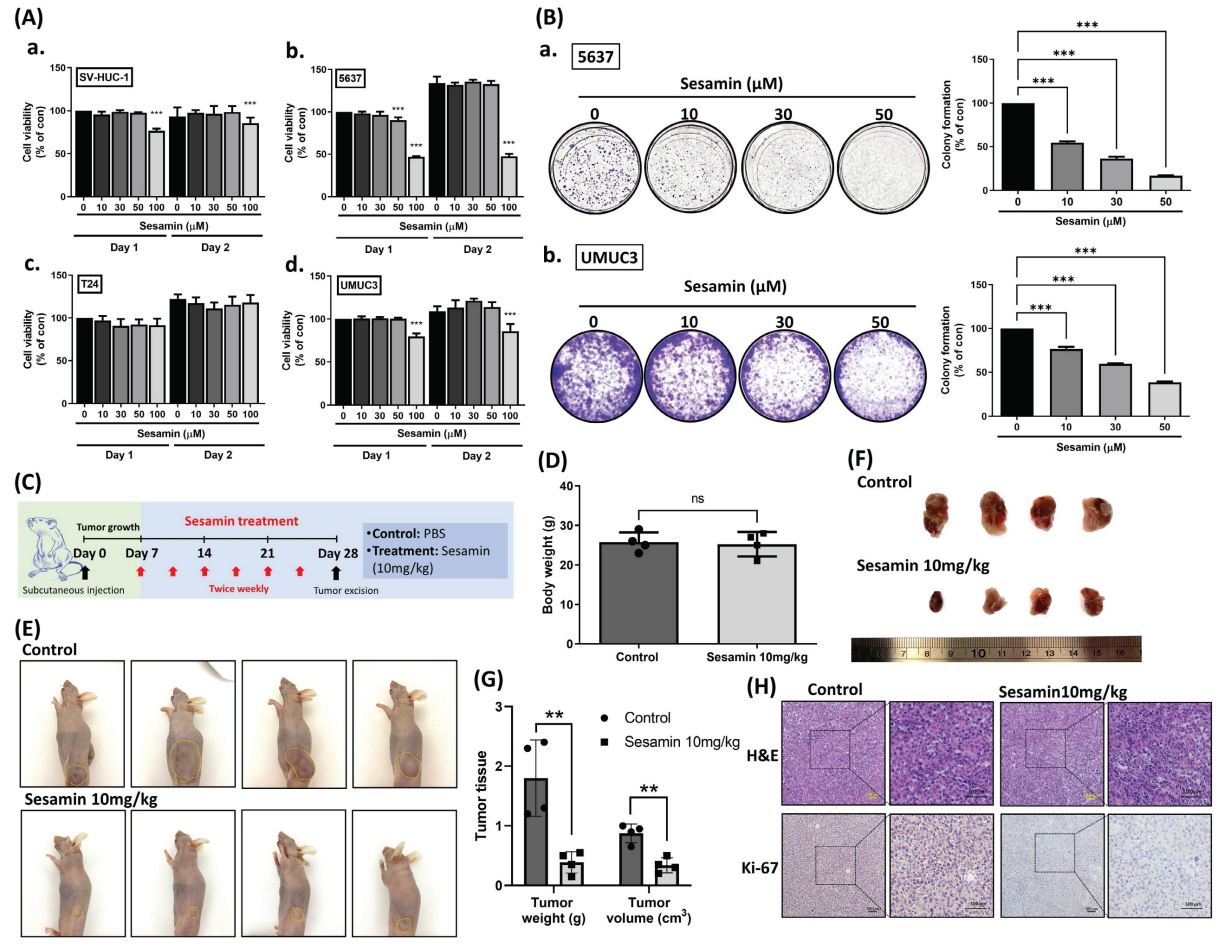
** Sesamin inhibits tumor growth in human BC.** (A) Treatment of human urinary tract epithelium cell (SV-HUC-1) and bladder cancer cell lines (T24, UMUC3, and 5637) with different concentration of sesamin (0, 10, 30, 50, 100 µM) for 24 h and 48 h. Cell viability was then measured by using a MTT reagent (n=3). (B) Colony formation assay was performed to detect cell survival following sesamin treatment at different concentrations (0, 10, 30, and 50 μM) for 7 days (n=4). (C) UMUC3 cells (1 x 10^6^) were subcutaneously inoculated into BALB/c nude mice. One-week post-injection, ensuring adequate tumor establishment, the mice were divided into two groups: a control group receiving PBS and a treatment group receiving intraperitoneal sesamin at 10 mg/kg, administered twice a week for three weeks. (D) The body weight of the mice in the control group and the sesamin treatment group (10 mg/kg) was recorded (n=4). (E-G) Tumor size for each mouse was captured in images, and tumor weight and volume were measured (n=4). (H) Histologic sections of mouse tumor tissues were stained with H&E, and IHC was performed to assess Ki-67 expression (n=4). All data are expressed as means ± SDs in triplicate samples. *P < 0.05, **P < 0.01, and ***P < 0.001 relative to the control group.

Firstly, we investigated the impact of sesamin on cell viability in normal human urinary tract epithelial cells (SV-HUC-1) and BC cells (5637, T24, UMUC3), all of which are muscle-invasive BC cells but differ in grade, with 5637 representing grade II and T24 and UMUC3 representing grade III. Cells were treated with varying concentrations of sesamin (0, 10, 30, 50, 100 μM) for two days. We observed that high concentrations of sesamin (100 μM) slightly reduced cell viability **(Fig. 1A)**. To further assess the long-term effects of sesamin, we conducted a colony formation assay. After one week, sesamin was observed to significantly diminish cell survival in BC cells in a dose-dependent manner **(Fig. 1B)**.

Next, we evaluated the efficacy of sesamin treatment in attenuating tumor growth by utilizing a mouse xenograft model **(Fig. 1C)**. Notably, without affecting body weight **(Fig. 1D)**, both tumor volume and weight were significantly reduced in the sesamin-treated group compared to the control group **(Fig. 1E-G)**. Furthermore, the expression of Ki-67, a proliferation marker, was markedly decreased in tumor tissues from the sesamin-treated group **(Fig. 1H; Supplementary Fig. 1A)**. These findings provide strong evidence for the potent anti-tumor effects of sesamin, demonstrating its ability to inhibit BC cell survival *in vitro* and suppress tumor growth *in vivo*.

### Sesamin inhibits cell migration, invasion, and resistance to anoikis in BC cells

**Figure 2:**
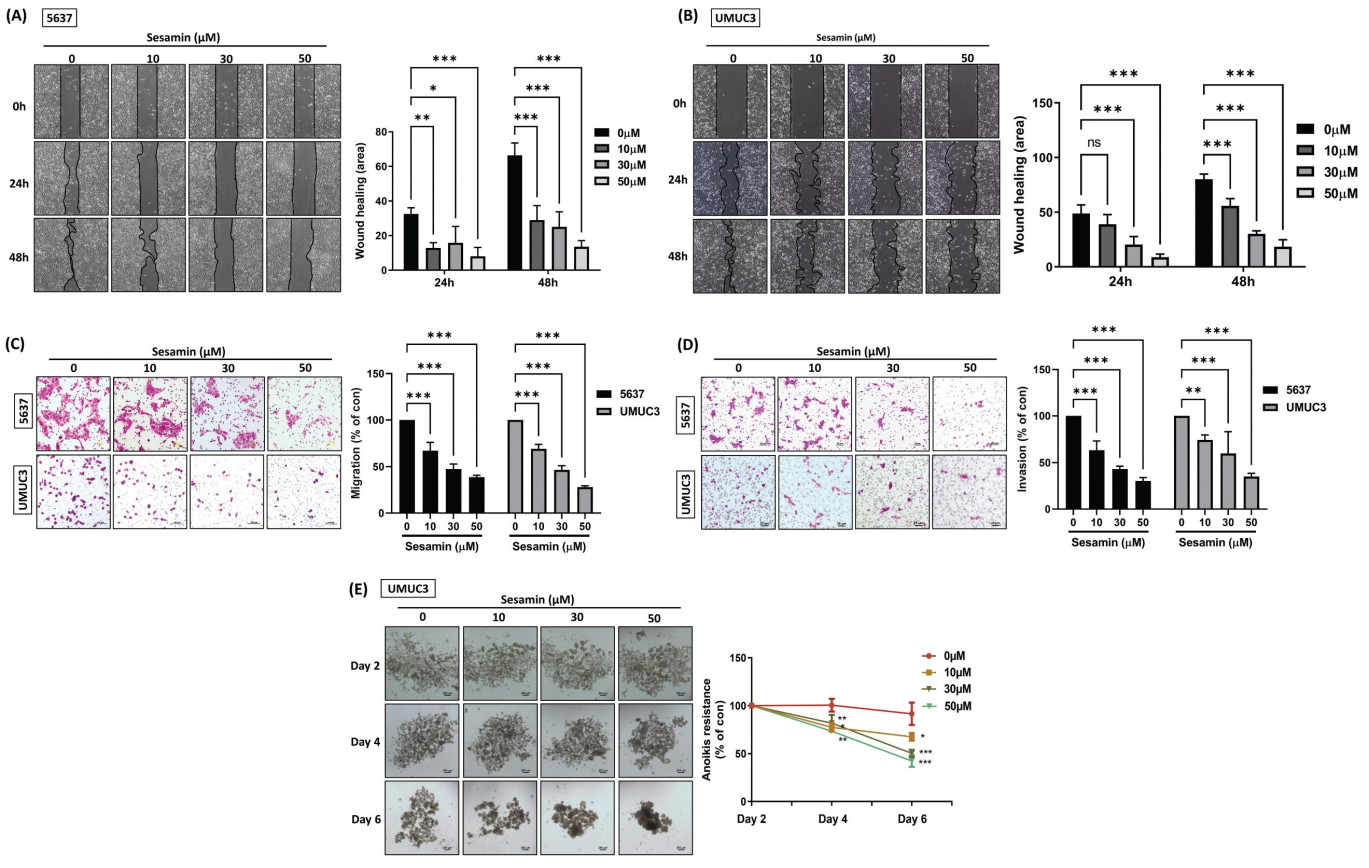
** Sesamin suppresses aggresiveness in human BC cells.** (A & B) BC cells were treated with varying concentrations of sesamin (0, 10, 30, and 50 μM) for 24 h, and a wound healing assay was performed to measure cell motility (n=3). (C & D) Transwell migration/invasion assay was conducted to analyze cell migration and invasion after 24 h of sesamin treatment at varying concentrations (0, 10, 30, and 50 μM) (n=3). (E) BC cells were incubated with varying concentrations of sesamin (0, 10, 30, and 50 μM) for 2, 4, and 6 days, and anoikis resistance was assessed (n=3). All data are expressed as means ± SDs in triplicate samples. *P < 0.05, **P < 0.01, and ***P < 0.001 relative to the control group.

Tumor progression encompasses a series of steps, including the coordination of cell proliferation, adhesion, migration, invasion, and angiogenesis 22. The potential of sesamin to inhibit the motility and invasiveness of BC cells remains unclear. Initially, the wound healing assay demonstrated that sesamin effectively inhibited BC cell motility **(Fig. 2A&B)**, a finding that was further supported by the Transwell migration assay **(Fig. 2C)**. Additionally, we employed the Transwell invasion assay, which showed that sesamin significantly suppressed BC cell invasiveness in a dose-dependent manner **(Fig. 2D)**. These results collectively indicate that sesamin inhibits both the motility and invasiveness of BC cells. Furthermore, since anoikis resistance is recognized as a critical early step in tumor metastasis 23, we investigated this aspect and found that sesamin effectively inhibited anoikis resistance in BC cells after 6 days of treatment **(Fig. 2E)**. Collectively, these findings suggest that sesamin holds potential as a therapeutic agent for inhibiting the malignant progression of BC by suppressing cell motility, invasiveness, and anoikis resistance.

### Sesamin suppresses levels of MMP2 production in BC cells

**Figure 3:**
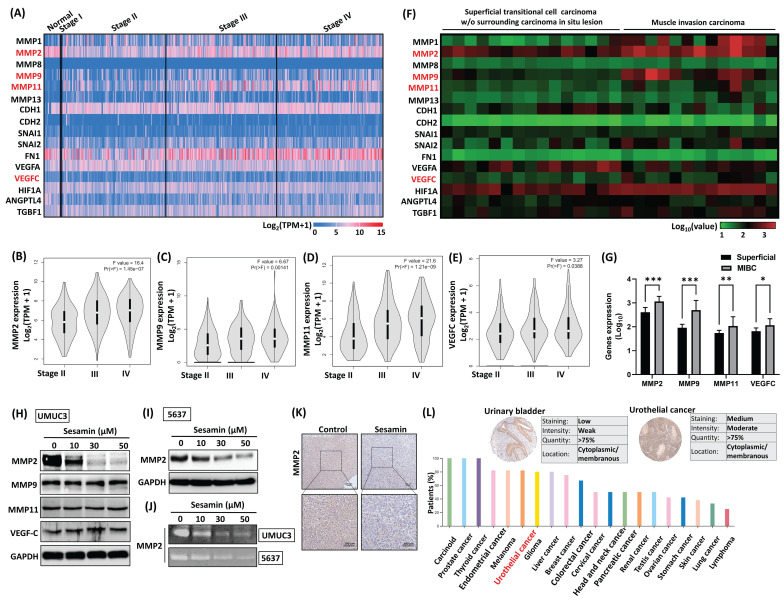
** Sesamin dose-dependently inhibits MMP2 protein expression in human BC.** (A-E) The expression levels of metastatic genes across different clinical stages were analyzed using the TCGA-BLCA and GEPIA2 web servers (normal, n=19; BC, n=412). The F value represents the F test. (F & G) The GEO dataset (GDS1479/ 201069_at) was used to analyze the expression of metastatic genes between superficial transitional cell carcinoma (n=15) and MIBC (n=12). (H) UMUC3 cells were treated with different concentrations of sesamin (0, 10, 30, and 50 μM) for 24 h, and the protein levels of MMP2, MMP9, MMP11, and VEGF-C were measured using a western blot assay (n=3). (I) MMP2 protein expression was measured by western blot assay following 24 h of sesamin treatment at varying concentrations (0, 10, 30, and 50 μM) in 5637 cells (n=3). (J) MMP2 activity was measured using a zymography assay (n=3). (K) IHC staining showed MMP2 expression in mouse tumor tissues (n=4). (L) MMP2 expression in human BC tissues and other tumor tissues was obtained from the Human Protein Atlas. All data are expressed as means ± SDs in triplicate samples. *P < 0.05, **P < 0.01, and ***P < 0.001 relative to the control group.

To understand the tumor-suppressive mechanisms of sesamin in BC, we identified genes consistently associated with key metastatic processes, including epithelial-mesenchymal transition, angiogenesis, and invasion pathways, ultimately selecting 16 metastatic hub genes. We then analyzed the expression levels of these 16 genes in BC tissues using data from the TCGA database **(Fig. 3A)**. As shown in **Figures 3B-E**, higher expression levels of MMP2, MMP9, MMP11, and VEGFC were positively correlated with advanced pathological stages. Similarly, GEO database analysis reveals that these 4 genes were more highly expressed in muscle-invasive bladder cancer (MIBC) compared to superficial BC **(Fig. 3F&G)**. However, treatment of BC cells with sesamin inhibited MMP2 expression without affecting MMP9, MMP11, or VEGFC **(Fig. 3H&I)**. Additionally, **Figure 3J** presents a representative gelatin zymogram showing a dose-dependent reduction in MMP2 gelatinolytic activity in BC cells. IHC staining analysis revealed that after sesamin treatment for three weeks, the expression of MMP2 decreased in the xenograft tumor tissues **(Fig. 3K; Supplementary Fig. 1B)**. Analysis of the Human Protein Atlas database discovered that MMP2 was highly expressed in human BC tissues, ranking fifth among 20 tumors **(Fig. 3L)**. These findings suggest that MMP2 is a promising therapeutic target and diagnostic biomarker in human BC, and sesamin may specifically suppress MMP2 expression, thereby reducing the malignant progression of the disease.

### STAT3 is involved in the inhibition of malignant progression by sesamin in BC

**Figure 4:**
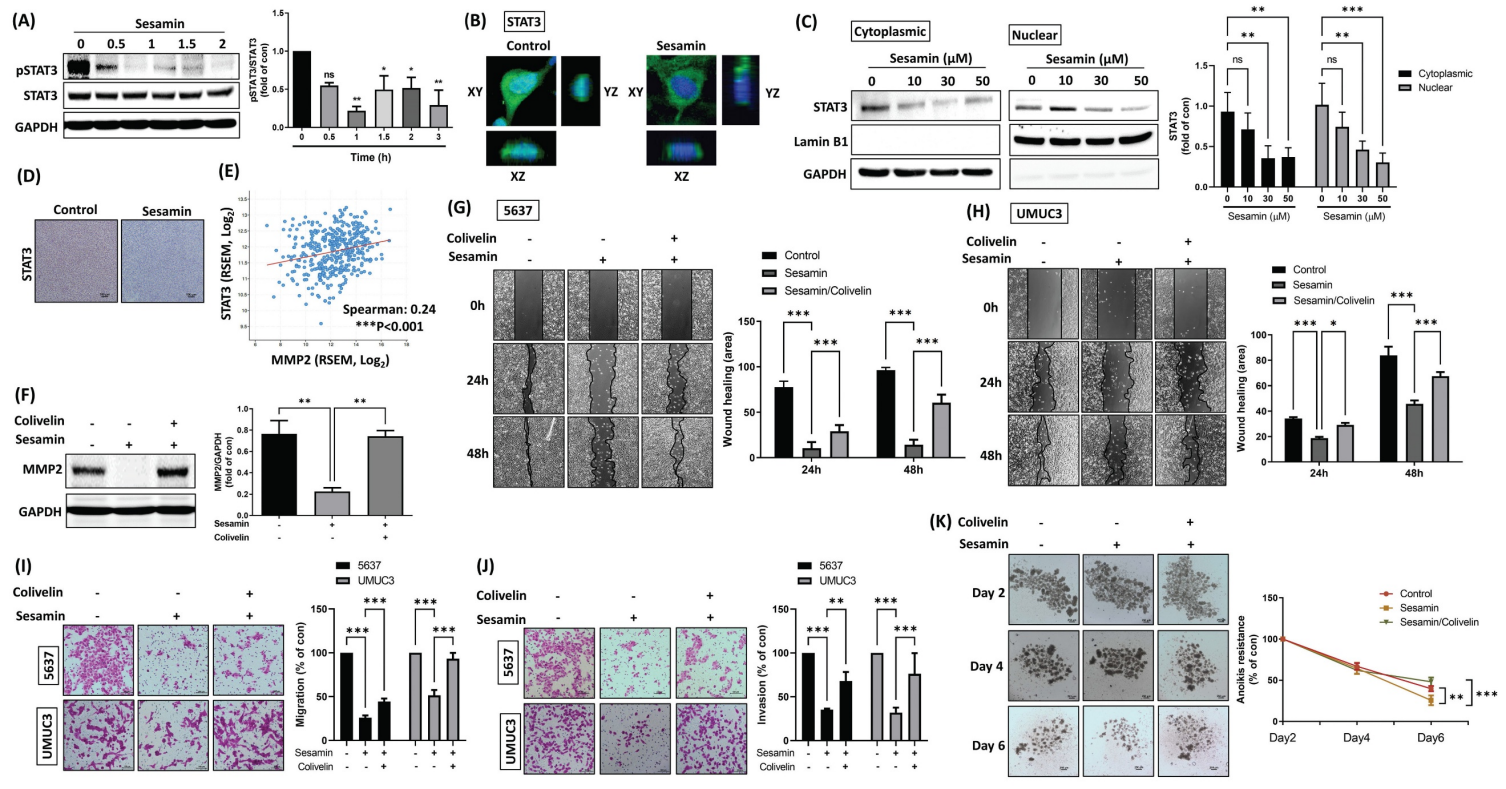
** STAT3 signaling molecule involvement in sesamin-inhibited aggressiveness in human BC.** (A) UMUC3 cells were treated with sesamin (50 mM) at different time intervals (0, 0.5, 1 1.5, 2 h), and the level of STAT3 phosphorylation was measured using a western blot assay (n=3). (B) STAT3 translocation from the cytoplasm to the nucleus was observed by confocal microscopy. STAT3 FITC-labeled antibodies (green), DAPI-stained DNA (blue) (n=3). (C) UMUC3 cells were treated with different concentrations of sesamin (0, 10, 30, and 50 μM) for 24 h, followed by the detection of STAT3 expression in the nucleus using a nuclear extraction assay (n=3). (D) IHC staining showed STAT3 expression in mouse tumor tissues (n=4). (E) The correlation between MMP2 and STAT3 mRNA expression in human BC tissues was analyzed using the TCGA-BLCA database (n=412). (F) UMUC3 cells were pre-treated with the STAT3 activator colivelin (0.5 μM) for 30 min, followed by sesamin (50 mM) treatment for 24 h. MMP2 protein levels were then measured using a western blot analysis. (G-K) BC cells were exposed to the STAT3 activator colivelin (0.5 μM) for 30 min prior to a 24 h treatment with sesamin (50 μM). Wound healing (n=3), Transwell migration/invasion (n=3), and anoikis resistance assays (n=4) were performed to evaluate the anti-tumor effects of sesamin in BC cells. All data are expressed as means ± SDs in triplicate samples. *P < 0.05, **P < 0.01, and ***P < 0.001 relative to the control group.

STAT3, a transcription factor, plays a crucial role in cancer by promoting tumor growth and progression, highlighting the importance of targeting STAT3 signaling in therapeutic interventions 24, 25. The resulting data revealed that sesamin variably inhibited STAT3 phosphorylation, with the most significant inhibitory effects observed at 1 and 3 h **(Fig. 4A)**. Treatment of BC cells with sesamin inhibited STAT3 translocation from the cytoplasm to the nucleus, as demonstrated by confocal imaging** (Fig. 4B)** and nuclear extraction assays **(Fig. 4C)**. IHC staining analysis revealed that sesamin treatment significantly reduced the expression of STAT3 in xenograft tumor tissues **(Fig. 4D; Supplementary Fig. 1C)**, indicating that the inhibitory effect of sesamin on STAT3 expression is consistently validated in both *in vitro* and *in vivo* experiments.

Based on the aforementioned results indicating the downregulation of phosphorylated STAT3, we employed a STAT3 activator (colivelin) to confirm whether sesamin attenuates MMP2 expression, cell migration, invasion, and anoikis resistance through STAT3 inhibition in BC cells. Initially, a negative correlation between MMP2 and STAT3 mRNA expression was observed in human BC tissues **(Fig. 4E)**. Pre-treatment of BC cells with a STAT3 activator mitigated the sesamin-induced reductions in MMP2 expression **(Fig. 4F)**. The STAT3 activator also significantly counteracted the sesamin-induced reductions in cell motility, invasion, and anoikis resistance in BC cells **(Fig. 4G-K)**. Collectively, these findings suggest that sesamin reduces MMP2 expression via inhibition of the STAT3 signaling pathway, ultimately leading to a decrease in the malignant progression of BC.

### LincRNA-p21 mediates the inhibition of STAT3-driven BC malignant progression by sesamin

**Figure 5:**
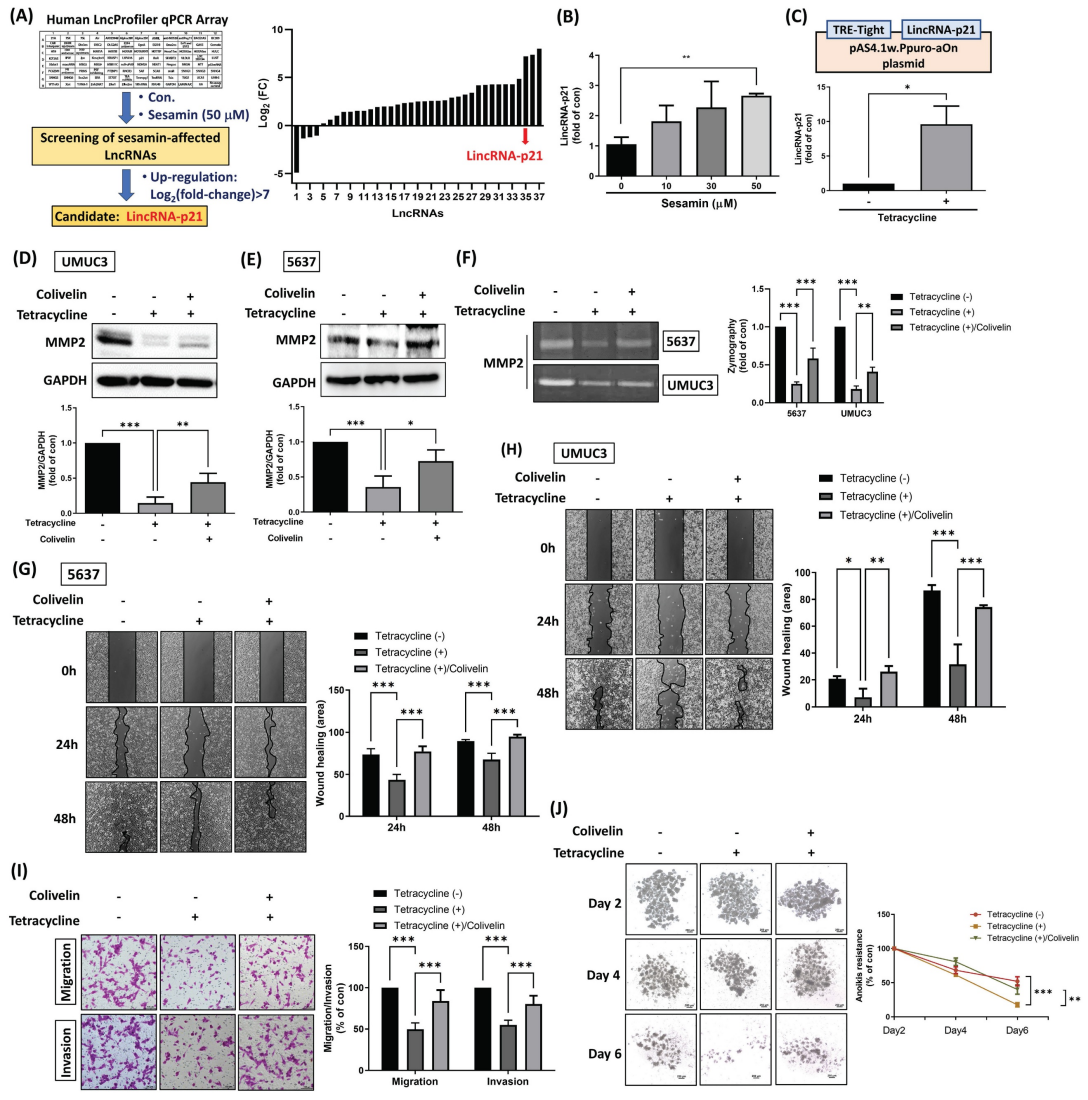
** Sesamin inhibits BC aggressiveness by upregulating lincRNA-p21.** (A) UMUC3 cells were treated with or without sesamin (50 μM) for 24 h, and a Human LncProfiler qPCR Array was used to assess LncRNAs expression. Define LncRNA expression as Log_2_(fold-change) > 7 to identify sesamin-affected LncRNAs in BC. (B) UMUC3 cells were treated with different doses of sesamin (0, 10, 30, and 50 μM) for 24 h, and lincRNA-p21 expression was detected by qRT-PCR assay (n=3). (C) LincRNA-p21 cDNA was cloned into the pAS4.1w.Ppuro-aOn plasmid. The plasmid (1μg/μL) was transfected into UMUC3 cells for 24 h, followed by treatment with tetracycline (10 μg/mL) for an additional 24 h. The levels of lincRNA-p21 expression were then measured using a qRT-PCR assay (n=3). (D-F) BC cells were co-administered with colivelin (0.5 μM) and tetracycline (10 μg/mL) for 24 h, and MMP2 protein expression and activity were measured by western blot assay and zymography, respectively (n=3). (G-I) Wound healing (n=3) and Transwell migration/invasion assays (n=3) were performed to evaluate cell movement and invasion of BC cells upon colivelin (0.5 μM) and tetracycline (10 μg/mL) co-treatment. (J) Anoikis resistance assay was conducted on days 2, 4, and 6 following co-treatment with colivelin (0.5 μM) and tetracycline (10 μg/mL) (n=3). All data are expressed as means ± SDs in triplicate samples. *P < 0.05, **P < 0.01, and ***P < 0.001 relative to the control group.

Numerous LncRNAs function as tumor suppressor genes, playing crucial roles in inhibiting cell proliferation, tumorigenesis, angiogenesis, and metastasis 26. To gain a deeper understanding of whether LncRNAs are involved in the sesamin-mediated inhibition of malignant progression in BC, a Human LncProfiler qPCR Array was employed to screen for LncRNAs affected by sesamin in BC cells. The analysis revealed a significant upregulation of lincRNA-p21, with a Log2(FC) > 7, in response to sesamin treatment **(Fig. 5A)**. Moreover, treatment with varying concentrations of sesamin resulted in a dose-dependent increase in lincRNA-p21 levels **(Fig. 5B)**.

Subsequently, lincRNA-p21 cDNA was cloned into the pAS4.1w.Ppuro-aOn plasmid to enable exogenous overexpression of lincRNA-p21 upon tetracycline treatment in BC cells **(Fig. 5C)**. Our findings indicated that tetracycline treatment results in a reduction of MMP2 protein expression and activity, as well as STAT3 activity; however, this effect was attenuated by co-treatment with a STAT3 activator **(Fig. 5D-F; Supplementary Fig. 2A&B)**, suggesting that lincRNA-p21 inhibits MMP2 expression through the downregulation of STAT3 signaling. Furthermore, tetracycline treatment significantly decreased cell motility, invasion, and anoikis resistance in BC cells, effects that were reversed by the STAT3 activator **(Fig. 5G-I)**. However, while tetracycline treatment also decreased cell survival, this effect was not rescued by the STAT3 activator, suggesting that the STAT3 signaling pathway is not involved in the regulation of lincRNA-p21-mediated suppression of cell survival **(Supplementary Fig. 3A&B)**. Taken together, these results suggest that lincRNA-p21 plays a critical role in the sesamin-mediated suppression of BC malignancy, primarily through the inhibition of STAT3 signaling and its downstream target--MMP2. This highlights the potential of lincRNA-p21 as a therapeutic tool in the management of human BC.

### Combination treatment with a PARP inhibitor and sesamin enhances chemosensitivity and reduces metastatic potential in BC

**Figure 6:**
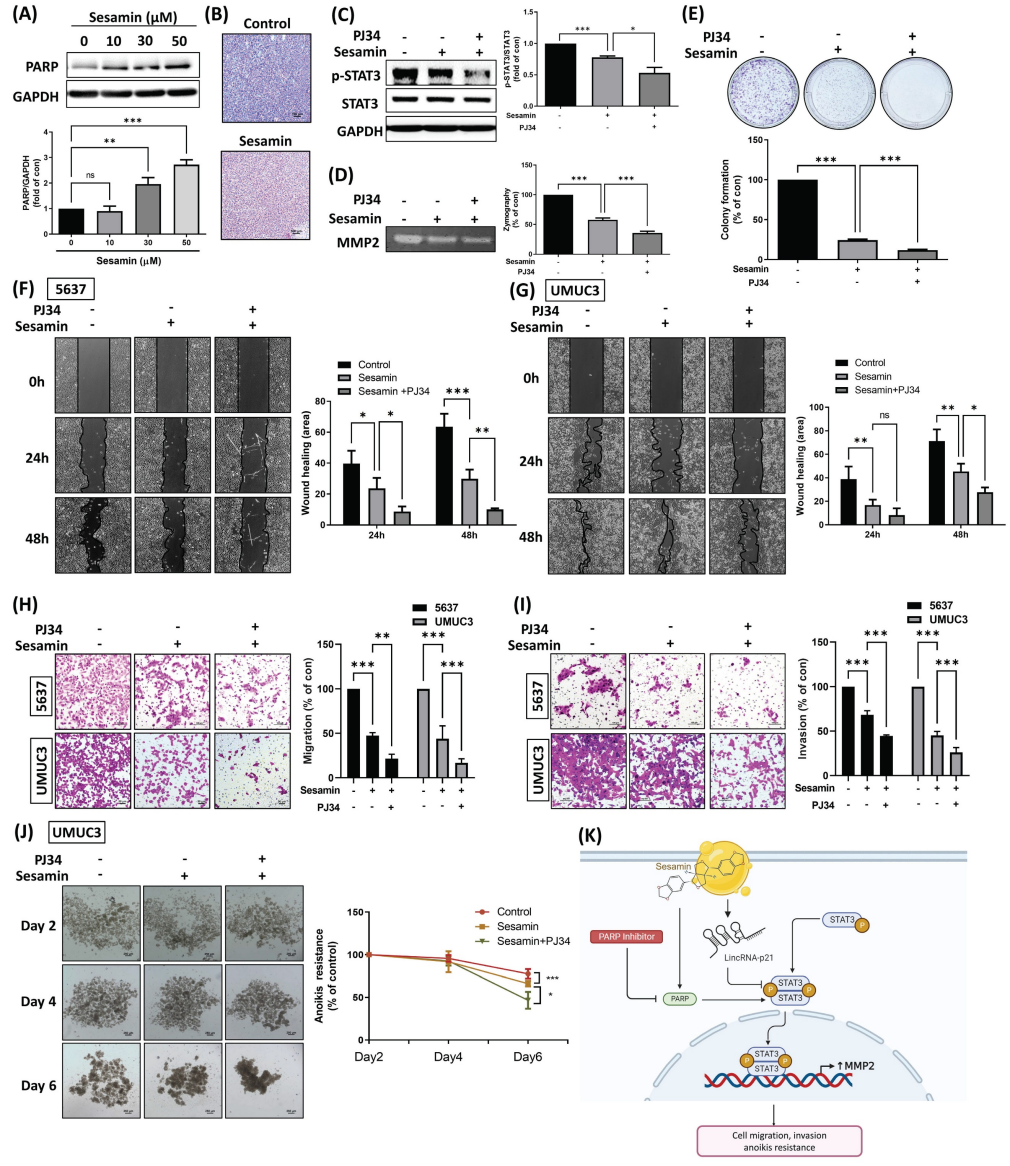
** The PARP inhibitor enhances the sesamin-mediated antitumor effects in human BC.** (A) UMUC3 cells were treated with different concentrations of sesamin (0, 10, 30, and 50 μM) for 24 h, and the protein levels of PARP weas measured using a western blot assay (n=3). (B) IHC staining revealed PARP expression in mouse tumor tissues (n=4). (C) UMUC3 cells were pre-treated with the PARP inhibitor (PJ34, 1 µM) for 30 min, followed by sesamin (50 µM) treatment for 24 h. The levels of STAT3 phosphorylation were measured by western blot assay. (D) Zymography assay was used to measure MMP2 activity (n=3). (E) Cell survival was assessed using a colony formation assay following co-treatment with PJ34 (1 μM) and sesamin (50 μM) for 7 days (n=4). (F-I) Wound healing (n=3) and Transwell migration/invasion assays (n=3) were performed to evaluate cell movement and invasion of BC cells upon PJ34 (1 μM) and sesamin (50 μM) co-treatment. (J) Anoikis resistance assay was conducted on days 2, 4, and 6 following co-treatment with sesamin (50 μM) and PJ34 (1 μM) (n=3). (K) The diagram shows how the sesamin, along with the PARP inhibitor, influences lincRNA-p21, which subsequently impacts STAT3 signaling. This signaling pathway ultimately leads to decreased MMP2 expression, inhibiting cell migration, invasion, and anoikis resistance in BC cells. All data are expressed as means ± SDs in triplicate samples. *P < 0.05, **P < 0.01, and ***P < 0.001 relative to the control group.

Sesamin has been reported to activate the DNA damage response and subsequently induce apoptosis in cancer cells 27. In the present study, PARP1, a highly sensitive sensor for DNA damage 28, was found to be induced upon sesamin treatment in BC cells **(Fig. 6A)**. IHC staining of PARP1 in xenograft tumor tissues showed consistent results, indicating that sesamin treatment activated PARP1 levels both* in vitro* and *in vivo* **(Fig. 6B; Supplementary Fig. 1D)**. We, therefore, hypothesize that PARP1 may have a protective effect on BC cells, potentially reducing the therapeutic efficacy of sesamin. To test this hypothesis, we used a novel and potent PARP inhibitor, PJ34, in the subsequent experiments. Interestingly, co-administration of sesamin and PJ34 further reduced STAT3 and MMP2 activity compared to sesamin treatment alone **(Fig. 6C&D)**. Moreover, co-treatment of BC cells with PJ34 significantly enhanced sesamin-induced reductions in cell survival, motility, invasion, and anoikis resistance **(Fig. 6E-J)**. The detailed mechanism shows that sesamin activates lincRNA-p21, which inhibits the phosphorylation and activation of STAT3, thereby reducing MMP2 expression and subsequently decreasing cell migration, invasion, and anoikis resistance. The use of a PARP inhibitor further blocks PARP activity, potentially amplifying the inhibitory effects of sesamin on STAT3 activation and MMP2 expression **(Fig. 6K)**. These findings suggest that targeting PARP1 in combination with sesamin may enhance its therapeutic effects in treating BC.

**Figure 7:**
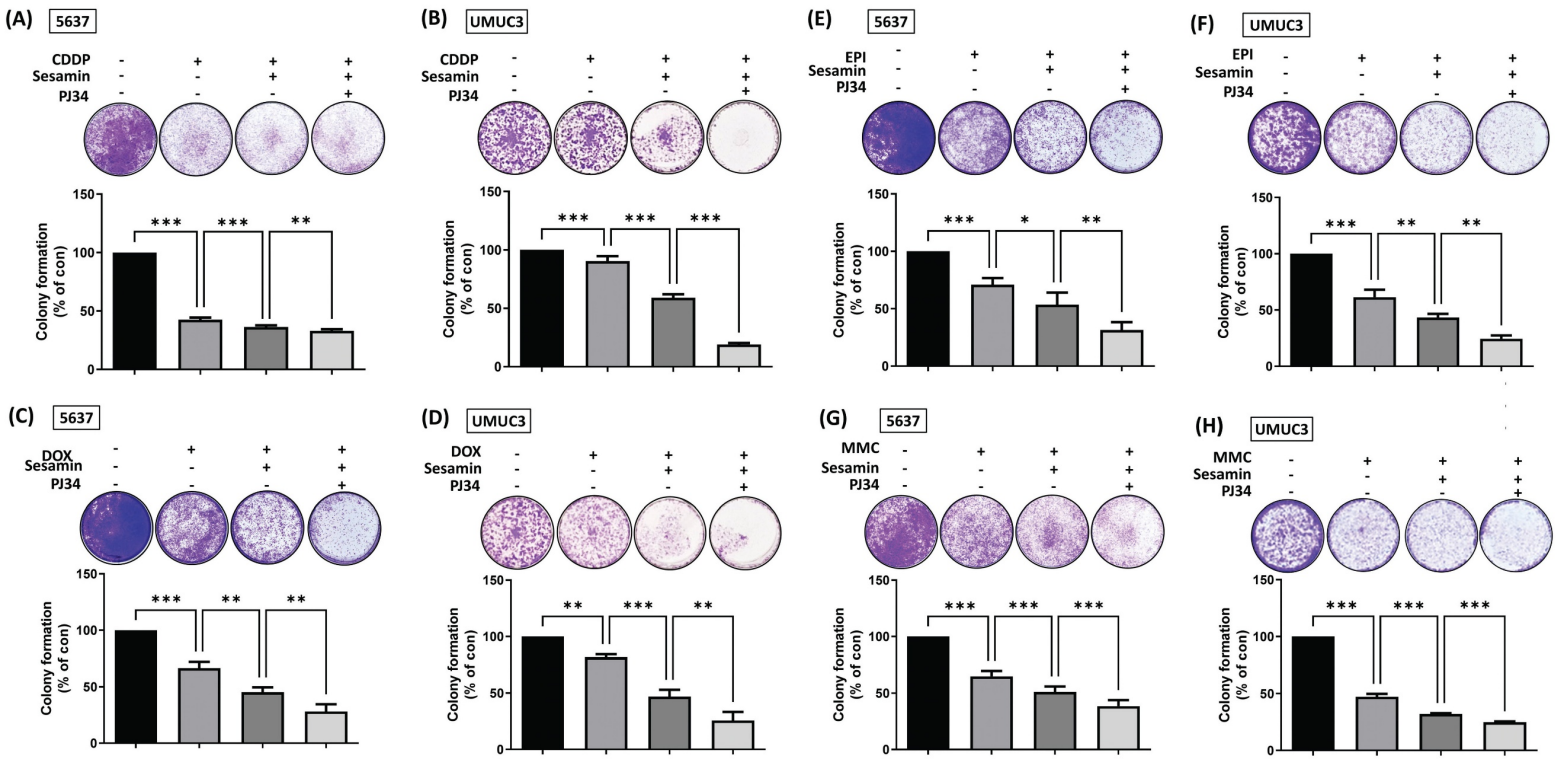
** Combination treatment of sesamin and PARP inhibitor enhances chemosensitivity in BC cells.** (A-H) BC cells were co-treated with sesamin (50 µM), PJ34 (1 µM), and chemotherapeutic drugs--cisplatin (CDDP, 2 µM), doxorubicin (DOX, 12.5 µM), epirubicin (EPI, 25 nM), or mitomycin-C (MMC, 50 nM). A colony formation assay was then performed over one week to assess cell survival (n=3). All data are expressed as means ± SDs in triplicate samples. *P < 0.05, **P < 0.01, and ***P < 0.001 relative to the control group.

Intravesical chemotherapy is a common treatment modality for BC, but its associated side effects can be significant 29, 30. To address this clinical challenge, we investigated whether the combination of sesamin and a PARP inhibitor, along with various chemotherapeutic agents, could enhance the chemosensitivity of BC cells. The resulting data showed that combining sesamin with chemotherapeutic agents, whether cisplatin (CDDP), doxorubicin (DOX), epirubicin (EPI), or mitomycin-C (MMC), enhanced the chemosensitivity of BC cells. Notably, the addition of a PARP inhibitor further optimized the effectiveness of the chemotherapy **(Fig. 7A-H)**. Taken together, these results suggest that the combination of sesamin, chemotherapeutic agents, and a PARP inhibitor could be a promising strategy for enhancing the efficacy of chemotherapy in BC treatment.

## Discussion

Sesamin, a lignan found in sesame seeds, can metabolically transform into two antioxidative metabolites, sesaminol and sesamolinol, within the liver of rats through enzymatic hydrolysis. Consuming sesamin may enhance liver health by boosting antioxidant defense mechanisms, suggesting potential health benefits of dietary sesamin 31. Evidence suggests that sesamin possesses beneficial properties, including the ability to modulate immune responses and reduce inflammation through mechanisms such as inhibiting inflammatory mediators and modulating signaling pathways 16. These findings highlight the therapeutic potential of sesamin in treating inflammatory diseases. Additionally, studies have shown that sesamin may offer benefits in cancer treatment, including inhibiting cell growth, reducing inflammation, preventing metastasis, and blocking tumor angiogenesis through various signaling pathways 32. Sesamin has shown promise as an agent for the potential prevention and treatment of colorectal cancer due to its ability to inhibit hypoxia-induced colorectal cancer angiogenesis through the NF-κB/HIF-1α/VEGFA signaling pathway, both *in vitro* and *in vivo* 33. Sesamin suppressed the proliferation of leukemic cells and triggered both apoptosis and autophagy by regulating caspase 3 and the mTOR/ULK1 signaling pathway, respectively 34. Sesamin directed the epithelial differentiation of cancer stem-like side population cells from gallbladder cancer, which is negatively associated with the NF-κB/IL-6/STAT3/Twist signaling pathway 35. In this study, we found that sesamin inhibits the malignant progression of BC, primarily by suppressing cancer cell proliferation, migration, invasion, and resistance to anoikis. Furthermore, combining sesamin with conventional chemotherapy drugs (cisplatin, doxorubicin, epirubicin, mitomycin-C) for BC can enhance chemotherapy sensitivity, highlighting the potential of sesamin as an adjuvant therapy for BC.

MMPs are responsible for modulating the dynamic remodeling of the ECM, and their increased expression is correlated with heightened cell proliferation and tumor size in various cancers 36-38. Elevated levels of MMP2 are correlated with a more malignant phenotype in melanoma and increased VEGF expression in gastric cancer 39, 40. MMP2 is also reported to be linked with poor prognosis in kidney clear cell carcinoma 41. A study using MMP2 knockout mice demonstrated a reduction in cell proliferation, neocapillary network growth, and migration of human umbilical vein endothelial cells in vitro, along with poor angiogenesis *in vivo* 42. Additionally, it is believed that inhibiting MMP2 can suppress angiogenesis and metastasis, and MMP2 has been considered a prognostic marker for predicting advanced stages and survival in BC 43, 44. In several clinical trials targeting MMP2, drugs such as Marimastat (BB-2516), Prinomastat (AG3340), and Tanomastat (BAY 12-9566) were designed to inhibit MMP2 activity with the aim of suppressing cancer progression 45. However, most of these trials demonstrated limited efficacy and were accompanied by significant side effects, particularly adverse musculoskeletal reactions 45. Given the notable side effects observed in current clinical trials involving MMP2 inhibitors, sesamin may present a novel approach with a more favorable safety profile.

Signaling molecules play a crucial role in regulating various cellular processes, including proliferation, apoptosis, and metastasis, making them promising targets for drug development in BC cancer treatment 46, 47. In BC cells, AKT signaling molecular was found to promote chemoresistance and trigger tumour growth 48, 49. Pretreatment of AKT inhibitor may suppress MMP2-induced BC cell migration and invasion 50. ETS2, a transcription factor, has been reported to activate MMP2 gene transcription in BC cells. Mechanistically, the overexpression of hsa-miR-146b stabilizes ETS2 mRNA, ultimately facilitating ETS2-induced MMP2 gene transcription in BC 51. It remains to be investigated whether sesamin suppresses MMP2 by inhibiting AKT activity or ETS2 expression in BC. On the other hand, STAT proteins are important transcription factors that regulate various cytokines and growth factors expression 52. Constitutively activated STAT3 has been found to promote tumorigenesis and increase the migratory ability in prostate epithelial cells 53. Moreover, the STAT3 signaling pathway contributes to several critical features of carcinogenesis and metastasis, such as increased cell proliferation and survival, along with enhanced migration and invasion 25, 54. Overexpression of STAT3 is associated with poor prognosis in hepatic, breast, thyroid, and gastric cancers 55. This study found that sesamin downregulates the expression and activity of MMP2, primarily through the STAT3 signaling pathway, thereby inhibits metastatic potential of BC cells. This indicates that sesamin inhibits the malignant progression of BC by targeting STAT3/MMP2 axis, highlighting its potential as a therapeutic agent for clinical treatment.

LincRNA-p21, which is regulated by p53 and HIF-1α, participates in a wide range of biological processes, such as controlling gene expression in both cis and trans, influencing mRNA translation, maintaining protein stability, modulating the Warburg effect, and facilitating p53-dependent apoptosis and cell cycle arrest 56, 57. LincRNA-p21 levels have been positively linked to the clinical response of breast cancer patients undergoing neoadjuvant chemotherapy. Enhancing lincRNA-p21 expression could serve as an effective approach to improve chemosensitivity in breast cancer patients with mutant p53 20. In head and neck squamous cell carcinoma (HNSCC), lincRNA-p21 is regulated at the transcriptional level by the mutant p53/nuclear transcription factor Y subunit alpha (NF-YA) complex. Reduced expression of lincRNA-p21 has been associated with more aggressive tumor progression *in vivo*, indicating its potential as a novel therapeutic target for HNSCC 58. Moreover, lincRNA-p21 suppresses BC cell growth by inhibiting glutaminase, reducing intracellular levels of glutamate and α-Ketoglutarate, and disrupting glutamine catabolism 59. This study is the first to demonstrate that sesamin enhances the expression of lincRNA-p21 in BC cells, enabling lincRNA-p21 to function as a tumor suppressor by inhibiting the metastatic potential of BC cells. Our findings suggest that lincRNA-p21 may serve as a promising therapeutic target for BC, offering new avenues for treatment strategies that aim to curb metastasis and improve patient outcomes. However, it is important to note that this study did not include animal model experiments to validate the functional role of lincRNA-p21 *in vivo*. Future studies will be essential to further confirm the therapeutic potential of lincRNA-p21 through *in vivo* animal models, which could provide more robust evidence for its clinical application in human BC.

PARP1 is known to have a significant positive correlation with the regulation of DNA repair 60. It is overexpressed in recurrent oral cancer, leading to high resistance of tumor cells to DNA damage therapies, indicating that PARP1 is part of a protective mechanism in cancer cells 61. Combining temozolomide with a PARP1 inhibitor (Olaparib) can enhance the sensitivity of temozolomide in IDH1-mutated gliomas 62. Recently, targeted therapies against PARP1 have been developed and approved for the treatment of BRCA-mutated breast, ovarian, and pancreatic cancers. Clinical trials have shown that PARP inhibitors can target tumors with homologous recombination deficiencies, such as BRCA-mutant cancers in breast cancer (NCT00494234) and ovarian cancer (NCT00753545) 63, 64. When combined with chemotherapy or radiation, PARP inhibitors significantly enhance efficacy in patients with recurrent or metastatic gastric cancer (NCT01063517) 65. In addition, PARP-1 regulates oncogenes, tumor suppressors, and inflammatory genes, providing therapeutic options for cancers driven by specific transcriptional factors, such as prostate cancer with ETS gene fusions (NCT01576172) 66. Beyond oncology, PARP-1 modulation has shown potential in reducing inflammation associated with stroke (NCT01983358) and myocardial infarction (NCT00271765) 67, 68. These applications highlight the broad clinical impact of PARP-1 across various diseases. This study reveals that sesamin induces PARP1 protein expression in BC cells in a concentration-dependent manner. When combined with a PARP1 inhibitor, sesamin significantly enhances its anticancer effects, including greater inhibition of BC cell proliferation, migration, invasion, and resistance to anoikis, as well as improved sensitivity to chemotherapy. These findings suggest that sesamin and PARP inhibitor combinations could represent a promising therapeutic strategy for overcoming chemoresistance in BC, potentially leading to better patient outcomes. Additionally, this approach may have broader implications for treating other cancers with PARP1 involvement, highlighting the translational potential of this combination therapy in clinical settings.

## Conclusions

**Figure 8:**
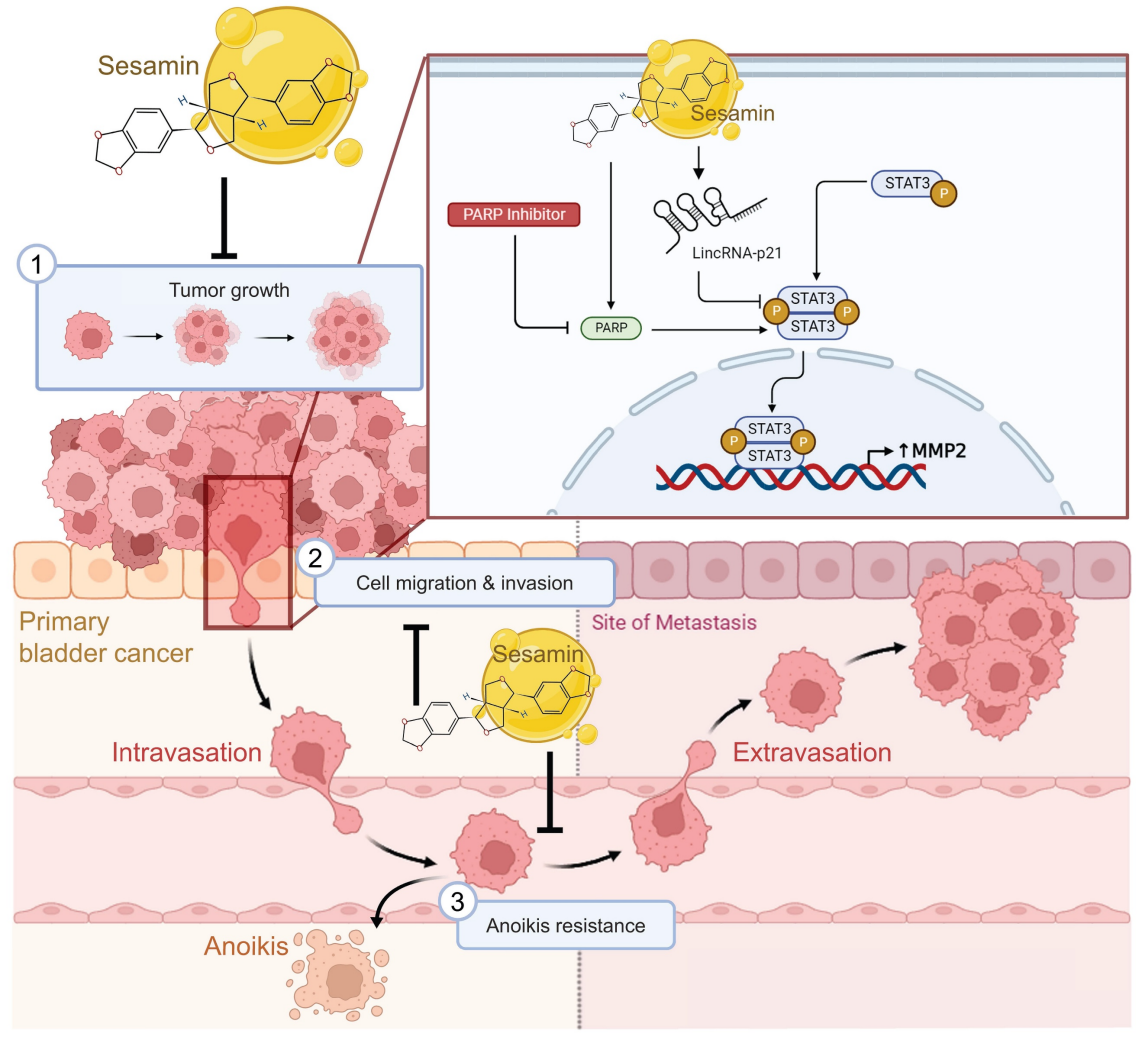
** Anticancer mechanisms of sesamin in human BC.** This illustration depicts the multifaceted anticancer effects of sesamin on BC. Sesamin reduces tumor growth (1), inhibits cell migration and invasion (2), and decreases resistance to anoikis (3), thereby impeding the metastatic spread of BC cells. Mechanistically, sesamin suppresses the expression of MMP2 by inhibiting the STAT3 signaling pathway, which is further regulated by lincRNA-p21. Additionally, the combination of sesamin with a PARP inhibitor enhances these inhibitory effects. These insights suggest that sesamin could serve as a promising therapeutic agent in clinical settings for the treatment of BC.

We elucidated the anticancer effects of sesamin, highlighting its multifaceted role in BC management. First, sesamin effectively prevents the formation of bladder tumors. Second, it exerts its antitumor effects by inhibiting MMP2 expression through the modulation of the lincRNA-p21/STAT3 signaling pathway, thereby reducing critical mechanisms of distant metastasis, including migration, invasion, and resistance to anoikis **(Fig. 8)**. Finally, our findings suggest that combining sesamin with a PARP inhibitor as an adjuvant therapy could enhance the efficacy of conventional chemotherapeutic agents, potentially allowing for reduced dosages and minimizing associated side effects. This underscores the promising potential of sesamin in improving therapeutic outcomes in BC.

## Supplementary Material

Supplementary materials and methods, figures.
