# Supplementary material for: Antitumor Effects of Sesamin via the LincRNA-p21/STAT3 Axis in Human Bladder Cancer: Inhibition of Metastatic Progression and Enhanced Chemosensitivity
Source: Int J Biol Sci. 2025 Mar 31;21(6):2692–706. doi: 10.7150/ijbs.103274 (PMC12035904; doi:10.7150/ijbs.103274)
Supplement: Supplementary file 1 — Supplementary materials and methods, figures. [file ijbsv21p2692s1.pdf]

Materials and Methods

Cell viability assay

Cell viability was assessed using the 3-(4,5-Dimethylthiazol-2-yl)-2,5-diphenyltetrazolium bromide (MTT) assay. SV-HUC-1, T24, UMUC63, and 5637 cells were plated at a density of $1x10^4$ cells per well in 96-well culture plates and allowed to adhere overnight. Cells were then treated with varying concentrations of sesamin (0, 10, 30, 50, and 100 μM) for 24 h, followed by incubating with MTT dye (0.5 mg/mL; Sigma-Aldrich, St. Louis, MO, USA) for 4 h at 37 °C in a CO_2 incubator. The supernatant was discarded, and the formazan crystals were dissolved in a specified volume of dimethyl sulfoxide (DMSO). The absorbance of the dissolved formazan was measured at 540 nm with a reference wavelength of 630 nm using a spectrophotometric plate reader.

Gelatin zymography

Gelatin zymography was employed to assess MMP2 activities. After treatment with sesamin at concentrations of 0, 10, 30, and 50 μM, serum-free medium was collected and mixed with 5x zymography sample buffer for 10 min. The samples were loaded onto 10% polyacrylamide gels containing 0.1% gelatin. Following electrophoresis, the gels were washed with 2.5% Triton X-100 and then incubated in activating buffer for 24 h. Subsequently, the gels were stained with 0.5% Coomassie Brilliant Blue R-250 for 30 min, followed by destaining (50% methanol: 10% acetic acid: 40% water). The protease activity appeared as clear bands against a dark blue background where the substrate had been digested. The densitometric analysis of these bands was performed using ImageJ software (National Institutes of Health, Bethesda, MD, USA).

Supplementary Figures

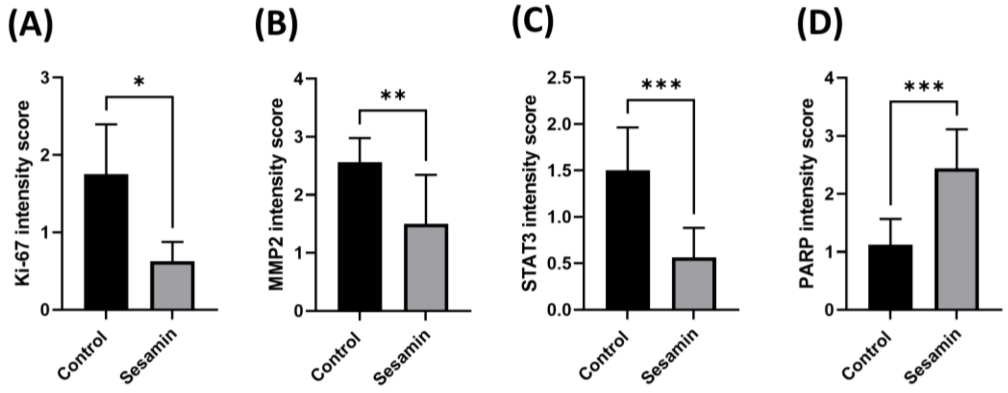

Supplementary Figure 1. Quantification of IHC staining results. (A–D) Quantitative IHC analysis of Ki-67, MMP2, STAT3, and PARP expression in mouse tumor tissues with or without sesamin treatment (N=4). All data are expressed as means ± SDs in triplicate samples. *P < 0.05, **P < 0.01, and ***P < 0.001 relative to the control group.

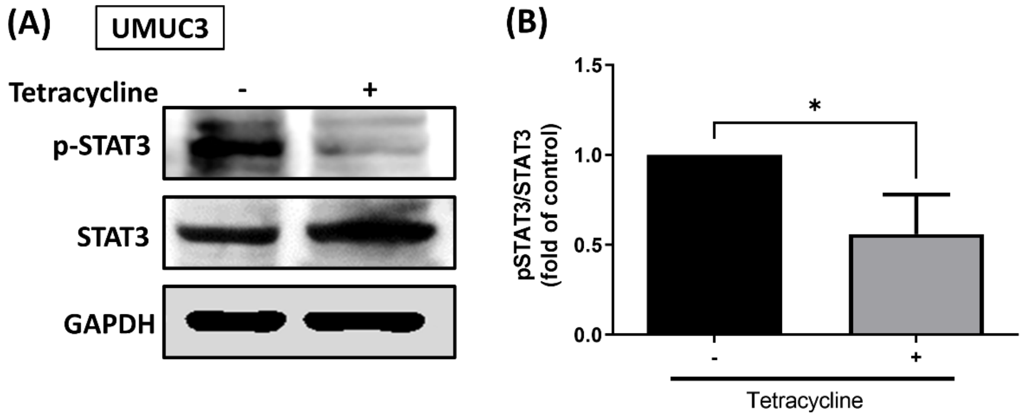

Supplementary Figure 2. LincRNA-p21 significantly inhibits STAT3 activity in BC cells. (A & B) The pAS4.1w.Ppuro-aOn plasmid containing LincRNA-p21 cDNA (1 μg/μL) was transfected into UMUC3 cells for 24 hours, followed by treatment with tetracycline (10 μg/mL) for an additional 24 hours. STAT3 phosphorylation levels were then measured using a western blot assay (n=3). All data are expressed as means ± SDs in triplicate samples. *$P < 0.05$ relative to the control group.

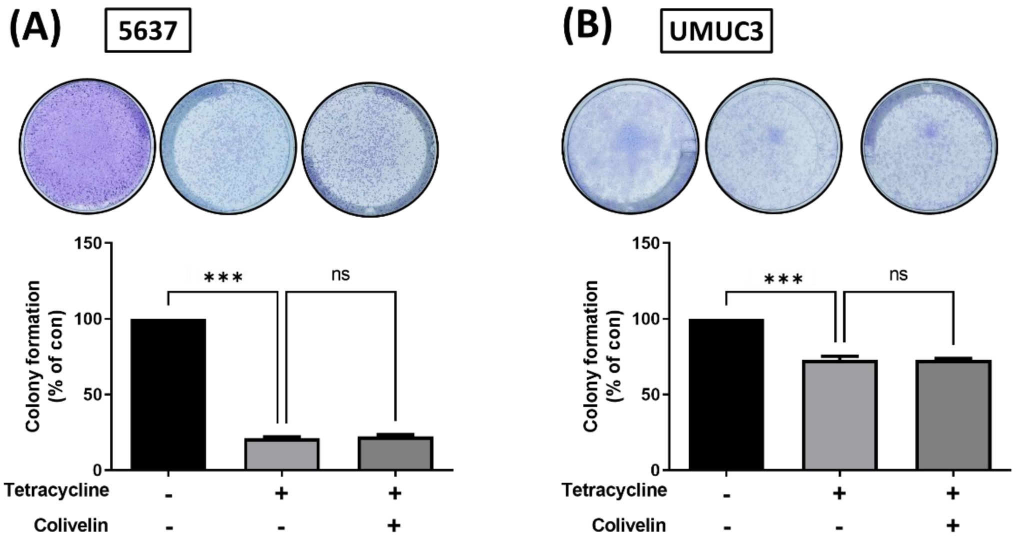

Supplementary Figure 3. The effect of the STAT3 signaling molecule on LincRNA-p21-mediated cell survival in BC cells. (A & B) A colony formation assay was performed to assess cell survival following co-administration of the STAT3 activator colivelin (0.5 μM) and sesamin (50 μM) for 7 days (n=3). All data are expressed as means ± SDs in triplicate samples. ***P < 0.001 relative to the control group.
